# Analysis of Serum Inflammatory Mediators Identifies Unique Dynamic Networks Associated with Death and Spontaneous Survival in Pediatric Acute Liver Failure

**DOI:** 10.1371/journal.pone.0078202

**Published:** 2013-11-11

**Authors:** Nabil Azhar, Cordelia Ziraldo, Derek Barclay, David A. Rudnick, Robert H. Squires, Yoram Vodovotz, for the Pediatric Acute Liver Failure Study Group

**Affiliations:** 1 Department of Surgery, University of Pittsburgh, Pittsburgh, Pennsylvania, United States of America; 2 Department of Computational and Systems Biology, University of Pittsburgh, Pittsburgh, Pennsylvania, United States of America; 3 Center for Inflammation and Regenerative Modeling, McGowan Institute for Regenerative Medicine, University of Pittsburgh, Pittsburgh, Pennsylvania, United States of America; 4 Department of Pediatrics, Washington University of St. Louis, St. Louis, Missouri, United States of America; 5 Department of Pediatrics, University of Pittsburgh, Pittsburgh, Pennsylvania, United States of America; Rutgers University, United States of America

## Abstract

**Background:**

Tools to predict death or spontaneous survival are necessary to inform liver transplantation (LTx) decisions in pediatric acute liver failure (PALF), but such tools are not available. Recent data suggest that immune/inflammatory dysregulation occurs in the setting of acute liver failure. We hypothesized that specific, dynamic, and measurable patterns of immune/inflammatory dysregulation will correlate with outcomes in PALF.

**Methods:**

We assayed 26 inflammatory mediators on stored serum samples obtained from a convenience sample of 49 children in the PALF study group (PALFSG) collected within 7 days after enrollment. Outcomes were assessed within 21 days of enrollment consisting of spontaneous survivors, non-survivors, and LTx recipients. Data were subjected to statistical analysis, patient-specific Principal Component Analysis (PCA), and Dynamic Bayesian Network (DBN) inference.

**Findings:**

Raw inflammatory mediator levels assessed over time did not distinguish among PALF outcomes. However, DBN analysis did reveal distinct interferon-gamma-related networks that distinguished spontaneous survivors from those who died. The network identified in LTx patients pre-transplant was more like that seen in spontaneous survivors than in those who died, a finding supported by PCA.

**Interpretation:**

The application of DBN analysis of inflammatory mediators in this small patient sample appears to differentiate survivors from non-survivors in PALF. Patterns associated with LTx pre-transplant were more like those seen in spontaneous survivors than in those who died. DBN-based analyses might lead to a better prediction of outcome in PALF, and could also have more general utility in other complex diseases with an inflammatory etiology.

## Introduction

Pediatric acute liver failure (PALF) is a complex, catastrophic, rapidly evolving clinical syndrome [1]. Like all complex diseases, the clinical trajectory of PALF is dynamic and non-linear. Its course reflects a complex interaction among the child's clinical condition, response to supportive care, disease severity, potential for recovery, and availability of a suitable organ if liver transplantation (LTx) is believed to be life-saving [2]. Yet, LTx is irreversible and impacts both society, in terms of organ allocation, as well as the individual patient and family coping with life-long immunosuppression and monitoring. Identification of patients likely to survive or die or whose condition would not benefit from LTx is necessary to inform LTx decisions.

Outcomes in PALF vary both between and among diagnostic categories, yet LTx occurs more commonly among those patients with an indeterminate diagnosis [3]. Recent data suggest immune or inflammatory dysregulation occurs in the setting of acute liver failure (ALF). For example, patients with acute liver failure (ALF) have increased risk for bacterial and fungal infections [4], aplastic anemia [5], [6], and impaired cell-mediated and humoral immunity [4]. Moreover, evidence of immune-inflammatory activation, characterized by marked elevation of soluble interleukin-2 receptor alpha (sIL-2R α), was identified in PALF [7]. These observations led us to hypothesize that immune or inflammatory dysregulation is present in PALF.

Acute inflammation elicits interrelated immune, inflammatory, neuronal, and physiological responses that can lead to severe organ dysfunction and death [8]. The complexity of the inflammatory response has stymied attempts at therapeutic modulation of acute inflammation. Computational modeling of complex systems is emerging as an approach to address the plethora of known and unknown interactions among biologic pathways [9], including those pathways operant in inflammation [10]. Recently, we used computational algorithms to assess multiple circulating inflammatory mediators coupled to dynamic network analyses to suggest both drivers and markers of inflammation in the setting of experimental trauma/hemorrhage in mice [11]. This methodology allowed us to identify unique cytokine interactions between mice undergoing trauma/hemorrhage compared to those who underwent sham intravenous cannulation procedure alone [11].

Our goal in the present study was to apply a similar methodology to PALF. Identification of immune/inflammatory networks will likely reflect dynamic changes in the inflammatory response and could lead to opportunities for directed therapeutic intervention, enhance liver transplant decisions, and improve patient outcomes.

## Materials and Methods

### PALF participants

This was a cohort study conducted through the Pediatric Acute Liver Failure Consortia (PALF; National Institutes of Health/National Institutes of Diabetes, Digestive, and Kidney Disease: 5U01 DK072146). Patients less than 18 years of age were eligible for enrollment into the PALF registry if they met the following entry criteria: 1) no known evidence of chronic liver disease, 2) biochemical evidence of acute liver injury, and 3) hepatic-based coagulopathy (not corrected with vitamin K) defined as a prothrombin time (PT)≥15 seconds or international normalized ratio (INR)≥1.5 in the presence of clinical hepatic encephalopathy (HE), or a PT≥20 seconds or INR≥2.0 regardless of the presence or absence of HE. During the period of this study, the PALF study group consisted of 22 pediatric sites: 19 centers in the United States, one in Canada, and two in the United Kingdom. Patient enrollment began in December 1999. The study was approved by the Institutional Review Boards from all of the participating institutions, and the NIH provided a Certificate of Confidentiality to the study (see *Materials S1* for full list). Written informed consent was obtained from the parents or guardians of the children in the study.

After enrollment into the PALF cohort, demographic and clinical data were recorded daily for up to seven days. Diagnostic evaluation and medical management were under the direction of the attending physician at each participating institution, and were consistent with the standard of care at each site. A final diagnosis for the cause of PALF was assigned by the primary physician at each study site as summarized previously [1]. 21-day outcomes were recorded as death without transplantation, LTx, or survival without LTx. A single daily serum sample was scheduled to be collected with the first morning blood draw following enrollment and daily for up to seven days, or until death, LTx, or discharge from hospital. The serum sample was divided into 250 µL or 500 µL aliquots, promptly frozen at -80°C at the enrollment site and later batch-shipped to the research bio-repository long-term storage. The frequency and volume of serum that could be collected for research purposes was dependent upon patient weight, hemoglobin, and the daily volume of blood required for diagnosis and patient management. Given these patient safety restrictions, research samples were not available at all potential time points for all PALF cohort participants.

A study cohort for this analysis was selected from the PALF cohort. We identified a convenience sample of participants to serve as the study cohort. Participants in the study cohort were to have at least 3 daily samples with at least 100 µL of serum. From those participants, the study cohort was further prioritized to capture those with the most samples available between study entry and outcome, and to recapitulate the diversity of age, diagnosis and outcome throughout the PALFSG as a whole.

### Assays of inflammatory mediators

We chose chemokines, cytokines, and reactive nitrogen oxide species that serve generally or specifically as biomarkers for various phases of the complex inflammatory response (see *Table S1*) : Cytokines and chemokines (eotaxin, granulocyte-macrophage colony stimulating factor [GM-CSF], interferon [IFN]-α2, IFN-γ, interleukin [IL]-1β, IL-1 receptor antagonist [IL-1ra], IL-2, soluble IL-2 receptor α chain [sIL-2rα], IL-4, IL-5, IL-6, IL-7, IL-8, IL-10, IL-12p40, IL-12p70, IL-13, IL-15, IL-17, IFN-γ-inducible protein of 10 kDa [IP-10; CXCL10], monocyte chemotactic protein-1 [MCP-1; CCL2], monokine induced by γ-interferon [MIG; CXCL9], macrophage inflammatory protein [MIP]-1α, MIP-1β, and tumor necrosis factor [TNF]-α) were assayed using a Luminex™ 100 IS apparatus (Luminex™, Austin, TX) using specific beadsets (Millipore, Billerica, MA). The nitric oxide reaction products NO_2_^−^+NO_3_^−^ were assayed using the nitrate reductase method (Cayman Chemical, Ann Arbor, MI).

### Statistical analyses

Patients' gender and coma grade at enrollment are reported as percentages. Outcomes are reported at 21 days. Age at enrollment is reported as medians (25^th^ and 75^th^ percentiles). Pearson chi-square tests were used to test differences in proportions between those patients in this inflammatory study and those not in this study, but in the PALF registry cohort. Wilcoxon Rank-sums tests were used to test for differences in distributions of age between the two groups. P-values less than 0.05 were used to determine statistical significance.

### Patient-Specific Principal Component Analysis [PCA]

This analysis identified subsets of mediators that most strongly correlated with the inflammatory response trajectory of an individual patient using at least 3 samples for each patient. A PCA score was then calculated for each cytokine as detailed in *Materials S2*, summarizing the relative degree to which that cytokine contributed to the inflammatory response for that patient over time. The PCA scores were used to group participants using hierarchical clustering as described below. Resultant patient sub-groups were then cross-correlated with clinical outcomes: spontaneous survivor (SS), non-survivor with native liver (NS), or received LTx (LTx). This method was unbiased in that all the patients' data were subjected to PCA independent of outcome groups.

### Hierarchical clustering analysis

This analysis highlighted the natural variability, as well as any overlap, in inflammatory mediators from among SS, NS, and LTx PALF participants. The details of this analysis are provided in *Materials S2*. The calculation is performed by using the Bioinformatics Toolbox in Matlab® 7.6.0, and the code for this algorithm and an explanation of its use in the context of experimental trauma/hemorrhagic shock has been made available publicly [11]. This method is unbiased, and so the segregation of post-PCA data by hierarchical clustering was likewise unbiased.

### Dynamic Bayesian Network analysis

This analysis delineated the connectivity among circulating inflammatory mediators as a function of time, thereby describing a possible biomarker signature as well suggesting possible mechanisms by which the progression of the inflammatory response differs based on patient sub-group. In this analysis, time courses of unprocessed cytokine measurements (e.g., measurements were not converted to PCA or to fold change over baseline, or normalized in any other way) from each experiment were used as input for a Dynamic Bayesian Network (DBN) inference algorithm, implemented in Matlab® essentially as described previously for gene array data [12]. A number of leave-one-out inference procedures were performed on the merged data to obtain a measure of the robustness of inferred interactions. The details of this analysis are provided in *Materials S2*.

## Results

### PALF participants

There were 986 participants enrolled in PALF at the time of this analysis. Available samples in the bio-repository were dynamic in that participants with at least 3 available samples would have become available or unavailable depending upon new participant enrollment and withdrawal of samples for other ancillary studies. We identified 49 PALF participants that met criteria for our convenience sample. Demographics of the 49 participants included in this analysis are presented in Table 1. 216 serum samples were analyzed in 49 participants. All participants had at least 3 and not more than 7 samples for analysis. The median number of samples per patient was 4 (25^th^ and 75^th^ percentiles were 3 and 6, respectively). The number of participants with samples tested was 18 with 3 samples, 12 with 4, 6 with 5, 7 with 6 and 6 with 7 samples analyzed. 15 participants had available samples on each day from enrollment to outcome or over 7 days from enrollment. For the remainder with at least one missing sample, 3 participants had 4 missing samples, 4 participants had 3 missing samples, 12 participants had 2 missing samples, and 15 participants had only one missing sample.

**Table 1:** Demographics, final diagnosis and outcomes of the Study Cohort.

	Study Cohort
	(n = 49)
	N (%)
**Age at enrollment (years)**	
**Median**	7.9
**25%, 75%**	1.1, 15.1
**Male**	25 (51.0)
**Diagnosis**	
**APAP toxicity**	8 (16.3)
**Autoimmune hepatitis**	5 (10.2)
**Viral infection**	3 (6.1)
**Indeterminate**	26 (53.1)
**Other diagnoses**	7 (14.3)
**Coma grade at enrollment**	
**Not assessable**	3
**0-I**	37 (78.3)
**II–IV**	10 (21.7)
**21-day outcome**	
**Alive without LT**	27 (55.1)
**LT**	15 (30.6)
**Died without LT**	7 (14.3)

### Variability of circulating inflammatory mediators in PALF participants

**Figure 1:**
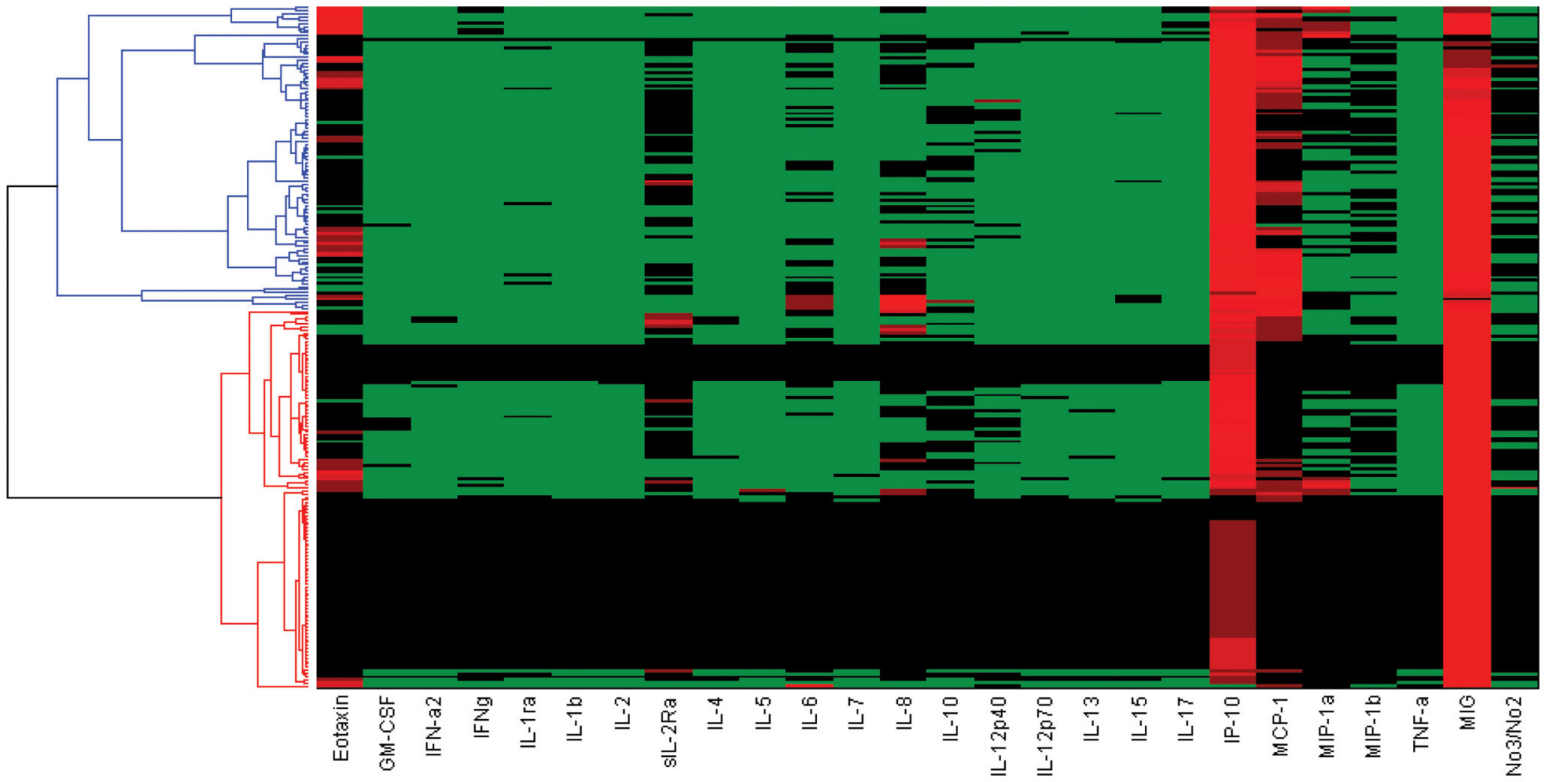
Hierarchical clustering of raw circulating inflammatory mediator data in PALF patients. Circulating inflammatory mediators in PALF spontaneous survivors, non-survivors, and LTx recipients were determined as described in the *Materials and Methods*. Unsupervised hierarchical clustering was performed as described in the *Materials and Methods*.

Plotting individual cytokine trajectories grouped by outcome did not reveal any obvious patterns or characteristic properties. (See Figs. S1, S2, S3). Hierarchical clustering was then used to segregate the cytokine data based on similar dynamic patterns to determine if participants with similar outcomes naturally clustered together based on their cytokine values, but concluded that unsupervised clustering of raw cytokine measurements was incapable of predicting clinical outcomes for these participants (see *Materials S2*). Indeed, the time courses of the 26 measured mediators were highly variable (Fig. 1; Figs. S1, S2, S3), and standard statistical analyses could not segregate among SS, NS, or LTx PALF participants (data not shown).

### Patient-Specific PCA separates some non-survivors from survivors and transplanted participants

**Figure 2:**
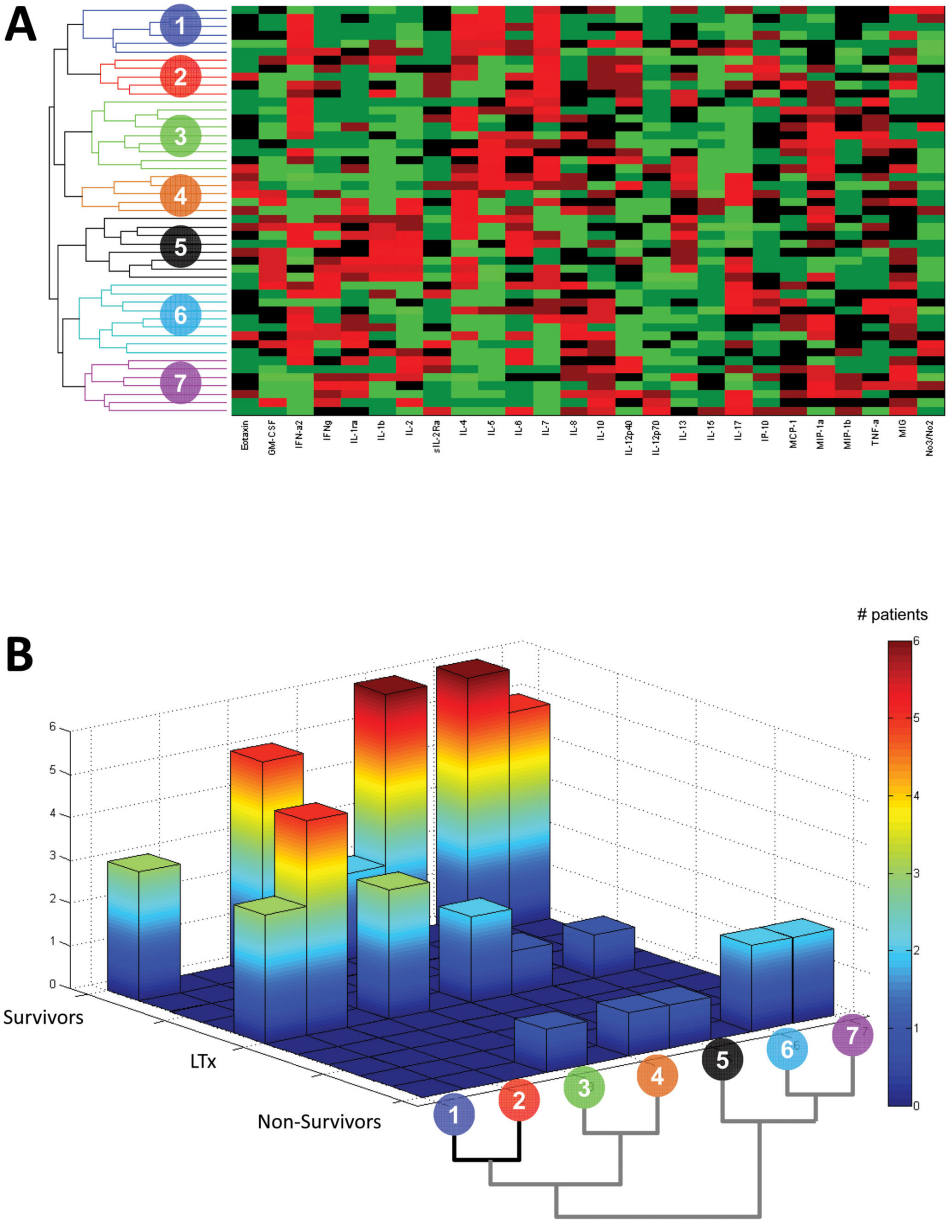
Hierarchical clustering of patient-specific PCA (“inflammation barcodes”) in PALF patients. The data from Figure 1 were subjected to patient-specific PCA (generating an “inflammation barcode”) followed by unsupervised hierarchical clustering as described in the *Materials and Methods*. *Panel A*: hierarchical clustering results, suggesting 7 distinct patient sub-groups. *Panel B*: Comparison of PALF sub-groups to “inflammation barcode”-defined sub-groups. Color spectrum bar represent the number of PALF patients.

We next utilized PCA to determine those circulating inflammatory mediators that dominated the overall patient-specific, time-dependent inflammatory profiles of the study cohort. We then performed hierarchical clustering on these individual inflammatory patterns and found that the participants segregated naturally into seven clusters (Fig. 2A). The first two of these clusters contained only survivors (Fig. 2B), suggesting that the inflammatory signature common to the participants in those clusters might be characteristic, and perhaps predictive, of spontaneous survival. We note that the PCA and clustering analyses are both unsupervised methods, and thus the algorithm was blinded with regard to outcome groups. After the clustering was performed, cluster assignments were cross-referenced to outcomes.

### Dynamic Bayesian Network analysis segregates PALF patient sub-groups

**Figure 3:**
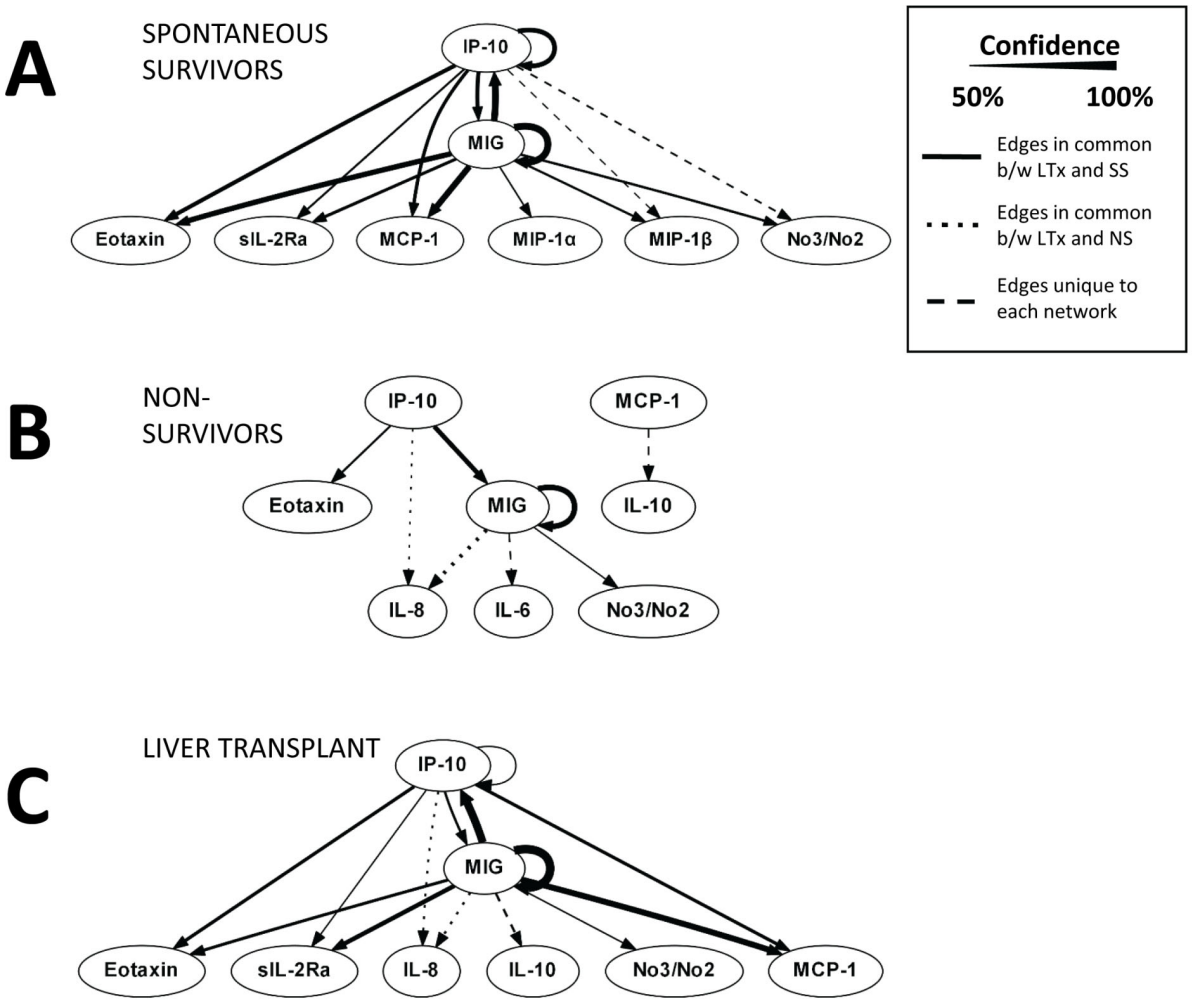
Dynamic Bayesian Network analysis of raw circulating inflammatory mediator data in PALF patients. The data from Figure 1 were subjected to DBN analysis as described in the *Materials and Methods*. Inflammatory mediators are shown as nodes, and the arrows connecting them suggest an influence of one mediator on the one(s) to which it is connected. The arrows do not distinguish positive from negative influences of one mediator on another. Semi-circular arrows suggest either positive or negative feedback of a given mediator on itself.

The PCA/hierarchical clustering analysis suggested common inflammatory signatures may be associated with clinical outcomes in the study cohort. We hypothesized that these inflammatory patterns could reflect dynamic inflammation networks that identify key inflammatory mechanisms in PALF. We therefore utilized Dynamic Bayesian Network (DBN) inference to determine if such networks could be discerned from the time courses of circulating inflammatory mediators in PALF participants. Results of this analysis on each of the three PALF sub-groups are shown in Fig. 3. Though the data were segregated by outcome group before being subjected to DBN inference, we note that the algorithm makes no assumptions about the connectivity of the network in any of the outcome groups. For SS: the DBN pattern suggested a network regulated via switching between the chemokines MIG/CXCL9 and IP-10/CXCL10, each of which drives its own expression and leads to the downstream production of eotaxin, sIL-2rα, MCP-1, MIP-1α, MIP-1β, and nitric oxide. In contrast, for NS: the DBN analysis suggested a primary network driven by the self-maintaining behavior of MIG/CXCL9, leading predominantly to the production of IL-6, IL-8, and nitric oxide. IP-10/CXCL10 was still present and driving eotaxin in this DBN, but without increasing its own production. A separate network consisting of MCP-1 driving IL-10 was also evident. For LTx: the DBN suggested a network very similar to that of spontaneous survivors, namely the apparent MIG/CXCL9 – IP10/CXCL10 switching with self-sustaining behavior and downstream production of eotaxin, sIL-2rα, IL-8, IL-10, MCP-1, and nitric oxide. To support the idea that the differences between groups did not arise by chance, we repeated the DBN analysis on a random grouping of the data and observed that the networks contained no major differences, with the core module of IP10/CXCL10 and MIG/CXCL9 cross-regulation and self-feedback being retained in all networks (Fig. S4)

## Discussion

The search for biomarkers in liver diseases has been fraught with difficulty. Putative biomarkers identified in small studies are often not sufficiently disease-specific, not tied mechanistically to a given disease, and non-reproducible in larger studies. Even those that are reproducible are frequently limited in their value to the most severe or advanced stages of disease [13]–[15]. Recent studies have emphasized the need for studying multiple inflammatory biomarkers combined with informatics/computational techniques as a key part of the interpretation of biomarker data [16]–[19]. This pilot study examines the use of novel analytic methods that may account for the dynamic complexity of the inflammatory response in PALF.

Analysis of raw circulating inflammatory mediator data in a convenience sample of PALF participants demonstrated a high degree of patient-to-patient variability. In contrast, when data underwent patient-specific PCA followed by unsupervised hierarchical clustering in a blinded fashion, seven distinct patient clusters were identified and some clusters contained only spontaneous survivors and LTx. This suggests that dynamic aspects of inflammation may be associated with patient-specific clinical outcomes. Finally, using DBN inference, cytokine networks associated with SS differed from those associated with NS. The cytokine network associated with LTx had characteristics more similar to SS than NS.

In a previous study on experimental trauma/hemorrhagic shock in genetically identical mice, we observed a high degree of variability in the raw circulating mediator data, but could identify distinct principal inflammatory drivers in animals receiving hemorrhagic shock from those subjected to sham cannulation [11]. In the present study, we affirm that the inflammatory response of a diverse cohort of PALF participants is highly variable [20]–[22] and that PCA could segregate inflammation patterns associated with different outcomes.

The inflammatory response can take two potential paths: (1) resolvable inflammation, in which the initial inflammatory response is harnessed by negative feedback that drives resolution, healing, and regeneration; or (2) unresolvable inflammation, which occurs when negative feedback is insufficient, a positive feedback loop of inflammation goes unchecked, and inflammation begets cellular damage and destruction which begets further inflammation [10], [23]–[27]. Positive and negative inflammatory feedback with regard to ultimate health status of the patient might be mapped onto pro- and anti-inflammatory mediators in some contexts, but not in others. Furthermore, any given mediator could serve a pro- or anti-inflammatory role at a given time or under given conditions. Thus, using data-driven models to suggest the time and context-dependent role of cytokines may be more informative than relying on an analysis with static, pre-assigned roles for cytokines. In a prior study, our data suggested that IP-10/CXCL10 initiates a low-level, resolvable inflammatory response in mice subjected to minor trauma alone. In contrast, MIG/CXCL9 drives a more robust and potentially unresolvable inflammatory response in the setting of the same minor trauma+hemorrhagic shock [11].

Several studies have demonstrated that both MIG/CXCL9 and IP-10/CXCL10 mRNA are elevated in experimental models of liver failure [28], [29], and circulating levels of both chemokines are implicated in chronic hepatitis C [30]. Importantly, and supporting our hypothesis that IP-10/CXCL10 may drive a resolvable inflammatory response in PALF, Bone-Larson *et al* demonstrated that IP-10/CXCL10 is hepatoprotective in an experimental model of acute liver injury [29]. Both MIG/CXCL9 and IP-10/CXCL10 are also associated with IFN-γ signaling, a pathway that also influences liver regeneration.

Based upon these preliminary studies, we hypothesized that if IP-10/CXCL10 is stimulated initially, then this chemokine would both drive its own production and suppress that of MIG/CXCL9 and lead to self-resolving inflammation. In contrast, if MIG/CXCL9 is stimulated initially, then MIG/CXCL9 would drive its own production while suppressing that of IP-10/CXCL10, in turn leading to self-maintaining, or unresolvable inflammation. Based on DBN analysis, we found what we suspect to be a pattern of resolvable inflammation associated with SS and unresolvable inflammation associated with NS in our selected PALF cohort. Specifically, we find PALF SS and LTx participant's exhibit chemokines MIG/CXCL9 and IP-10/CXCL10, each of which exhibits feedback behavior and each of which appears to regulate the other. This chemokine switching network appears to drive a diverse inflammatory response that includes eotaxin, sIL-2rα, MCP-1, MIP-1α, MIP-1β, and nitric oxide (in spontaneous survivors) or eotaxin, sIL-2rα, IL-8, IL-10, MCP-1, and nitric oxide (LTx recipients). Accordingly, we interpret the inflammation networks in PALF SS and LTx participants as demonstrating the possibility of switching between inflammatory responses driven by IP-10/CXCL10 vs. inflammation driven by MIG/CXCL9, and ultimately manifesting in a pattern of resolvable inflammation. In contrast, PALF NS participants exhibited an inflammation network that suggests feedback behavior for MIG/CXCL9 but not for IP-10/CXCL10, driving the production of IL-6, IL-8, and nitric oxide and resulting in unresolvable inflammation.

There are several limitations to our study. This was a retrospective study, and the identified networks were not validated in a separate cohort of participants. The study cohort, as well as sampling time points, was heterogeneous, though the participants in the study cohort were representative of the larger PALF Study Group cohort, and the time frame of sampling was restricted to 7 days post-enrollment. Only a subset of inflammatory mediators was assayed, and, importantly, damage-associated molecular pattern molecules (e.g. HMGB1) were not assessed.

In conclusion, the present study suggests that the DBN-defined inflammatory networks might serve as powerful, new biomarkers for predicting outcomes in PALF, which represents a novel use of DBN inference methodology. These findings will be validated in a larger patient cohort with sampling time points extending to the outcome of death, discharge, or LTx if that outcome was beyond seven days. Despite a heterogeneous inflammatory response in individual PALF participants, our studies suggest a network-based analysis may have the potential to segregate spontaneous survivors and non-survivors with LTx recipients having a biomarker pattern more similar to spontaneous survivors than those of non-survivors. Our data also leave open the possibility that these chemokine-based, feedback-driven inflammatory switching mechanisms might actually mediate such outcomes. This consideration, in turn, suggests that it might be possible to identify subsets of PALF patients in whom specific immune-modulatory therapy might improve the likelihood of spontaneous survival.

## Supporting Information

Figure S1 **Detailed time courses of circulating inflammatory mediators in PALF spontaneous survivors.** The data depicted in Figure 1 are shown as detailed time courses for each patient in the PALF spontaneous survivor sub-group. Values for all cytokines and chemokines are in pg/ml. Values for NO_2_^−^/NO_3_^−^ are in µM.
(TIF)

Figure S2 **Detailed time courses of circulating inflammatory mediators in PALF non-survivors.** The data depicted in Figure 1 are shown as detailed time courses for each patient in the PALF non-survivor sub-group. Values for all cytokines and chemokines are in pg/ml. Values for NO_2_^−^/NO_3_^−^ are in µM.
(TIF)

Figure S3 **Detailed time courses of circulating inflammatory mediators in PALF LTx recipients.** The data depicted in Figure 1 are shown as detailed time courses for each patient in the PALF LTx recipient sub-group. Values for all cytokines and chemokines are in pg/ml. Values for NO_2_^−^/NO_3_^−^ are in µM.
(TIF)

Figure S4 **DBN results from randomized outcome groups.** Patients were grouped randomly into three groups of sizes of 27, 15, and 7 while maintaining approximately the same percentage of SS (55%):NS (14%):LTx (31%) in each group (panel A) or allowing the percentages to vary (panel B). DBNs were inferred on each group and showed no major differences, with the core module of IP-10 and MIG self-feedback and cross-regulation being observed in all networks. Groups I, II, and III have 15, 27, and 7 patients respectively.
(PDF)

Table S1 **List of inflammatory mediators assayed in patient serum.**
(TIF)

Materials S1 **List of the Institutional Review Boards from all of the participating institutions.**
(DOCX)

Materials S2 **Detailed description of PCA, hierarchical clustering, and DBN methods.**
(DOCX)
