# Supplementary figures and images for: Analysis of Serum Inflammatory Mediators Identifies Unique Dynamic Networks Associated with Death and Spontaneous Survival in Pediatric Acute Liver Failure
Source: PLoS One. 2013 Nov 11;8(11):e78202. doi: 10.1371/journal.pone.0078202 (PMC3823926; doi:10.1371/journal.pone.0078202)

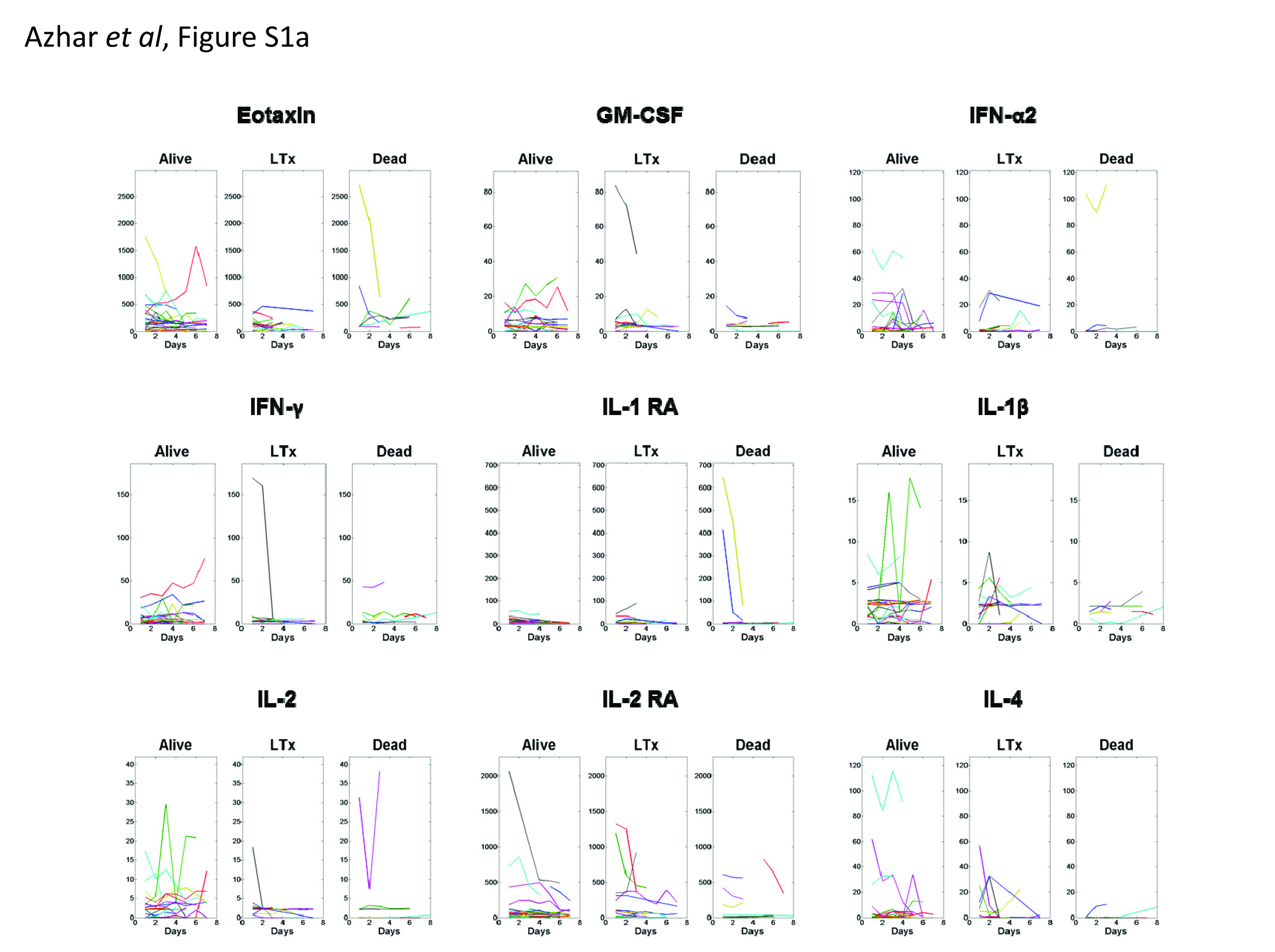

Supplement: Figure S1 — Detailed time courses of circulating inflammatory mediators in PALF spontaneous survivors. The data depicted in Figure 1 are shown as detailed time courses for each patient in the PALF spontaneous survivor sub-group. Values for all cytokines and chemokines are in pg/ml. Values for NO2 −/NO3 − are in µM. (TIF) [file pone.0078202.s001.tif]

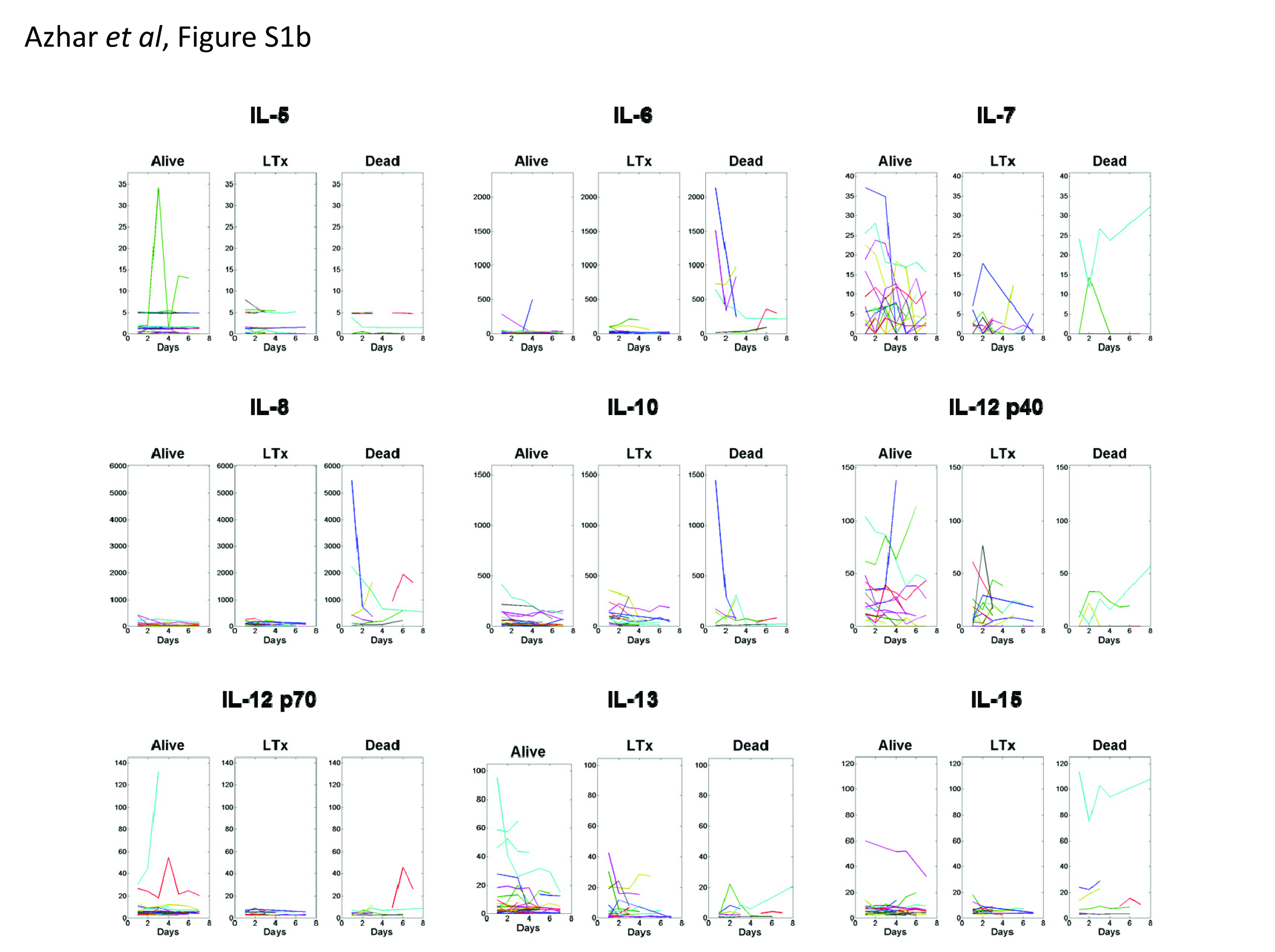

Supplement: Figure S2 — Detailed time courses of circulating inflammatory mediators in PALF non-survivors. The data depicted in Figure 1 are shown as detailed time courses for each patient in the PALF non-survivor sub-group. Values for all cytokines and chemokines are in pg/ml. Values for NO2 −/NO3 − are in µM. (TIF) [file pone.0078202.s002.tif]

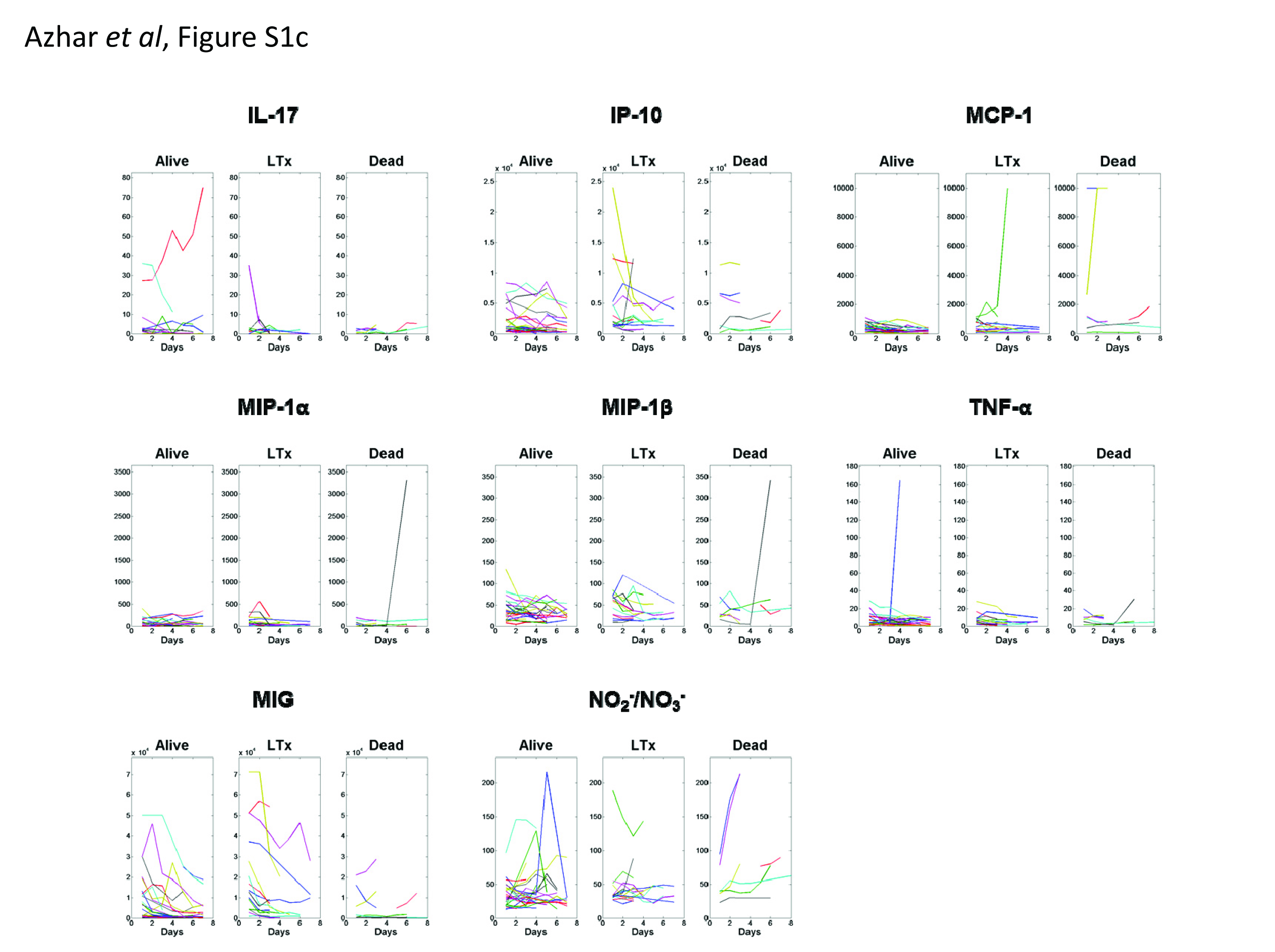

Supplement: Figure S3 — Detailed time courses of circulating inflammatory mediators in PALF LTx recipients. The data depicted in Figure 1 are shown as detailed time courses for each patient in the PALF LTx recipient sub-group. Values for all cytokines and chemokines are in pg/ml. Values for NO2 −/NO3 − are in µM. (TIF) [file pone.0078202.s003.tif]

**A**

Repeat 1

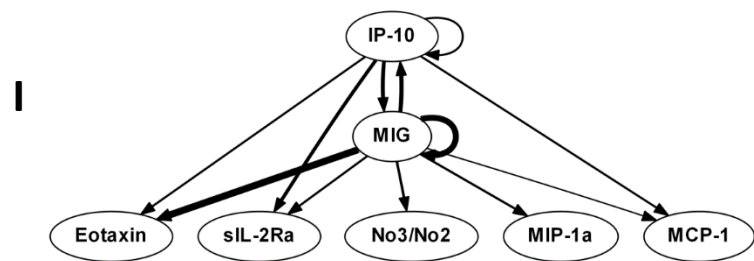

II

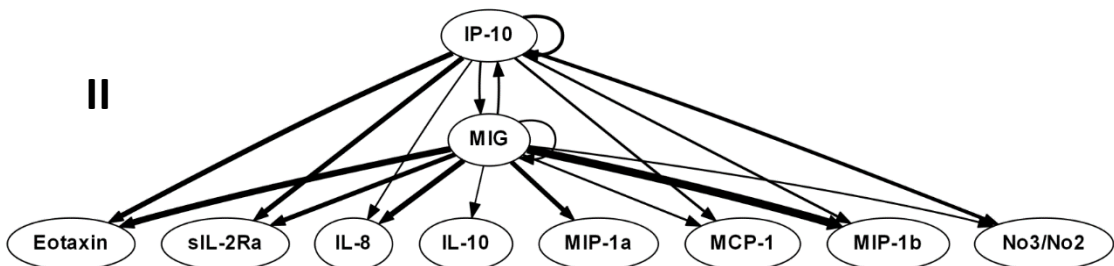

III

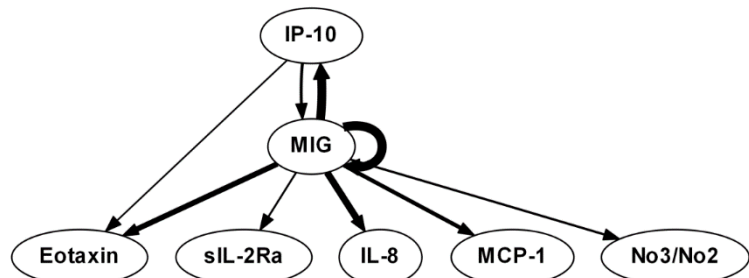

Repeat 2

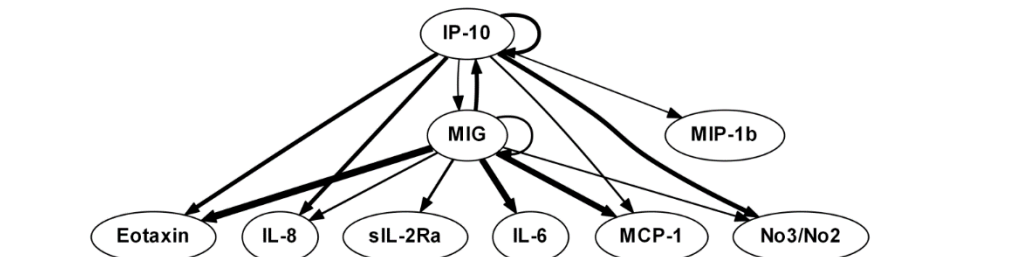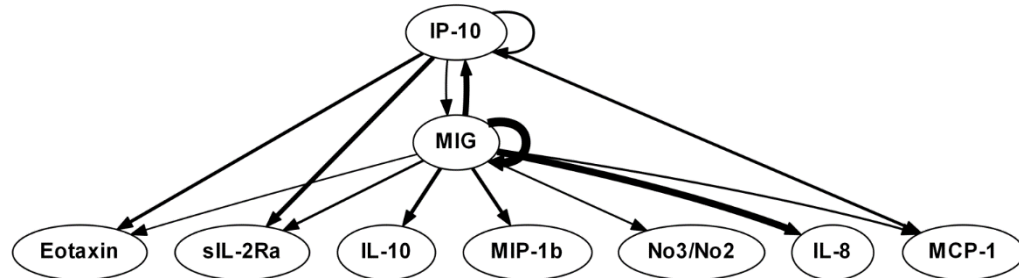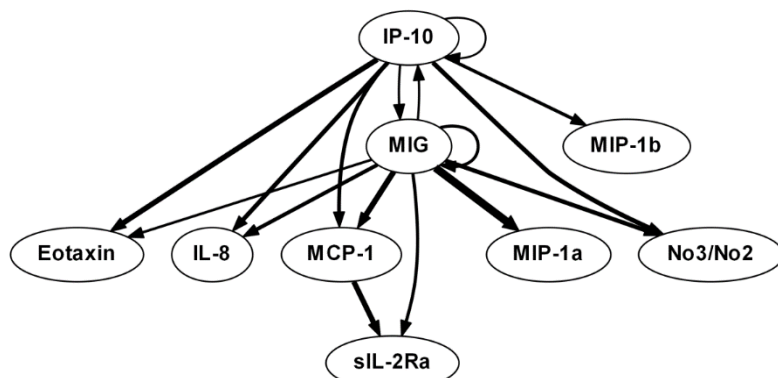

Repeat 3

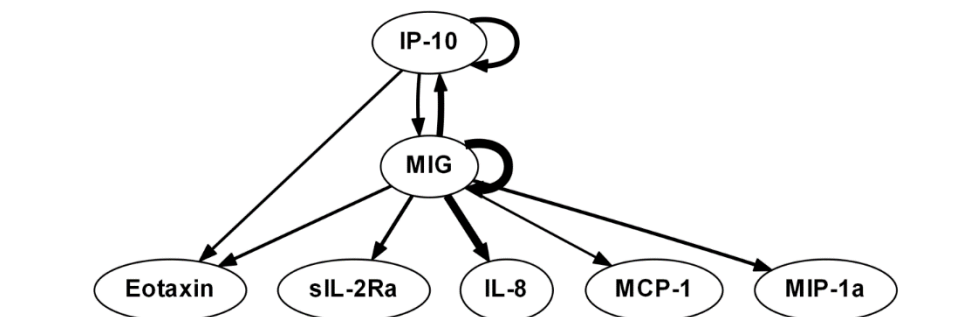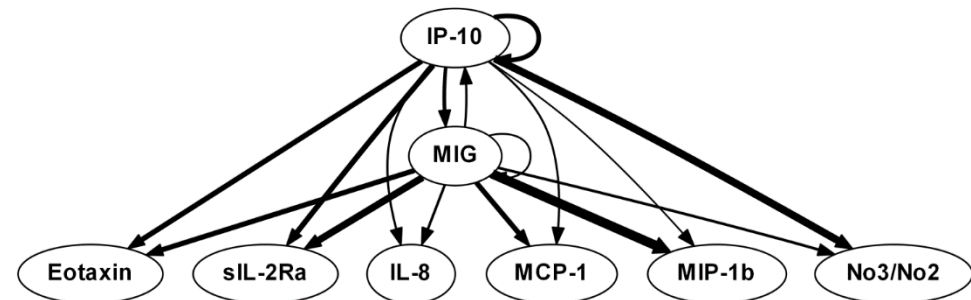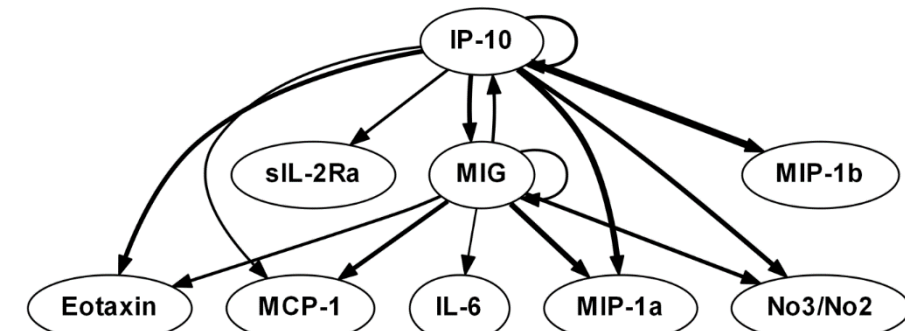**B**

Repeat 1

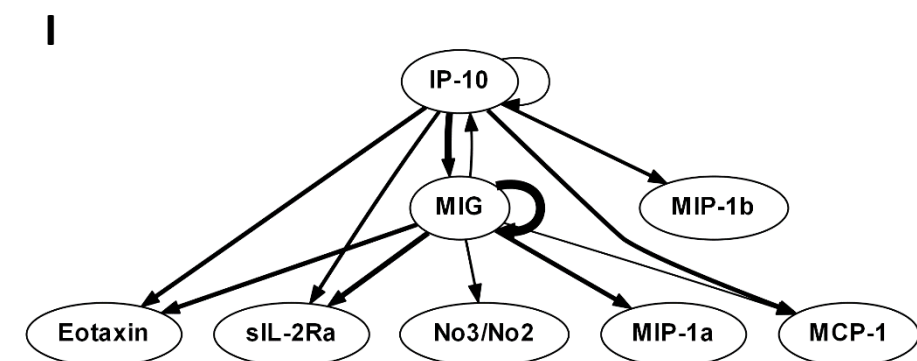

II

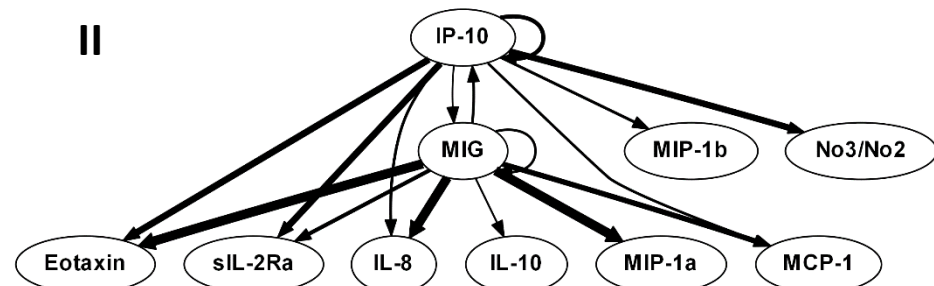

III

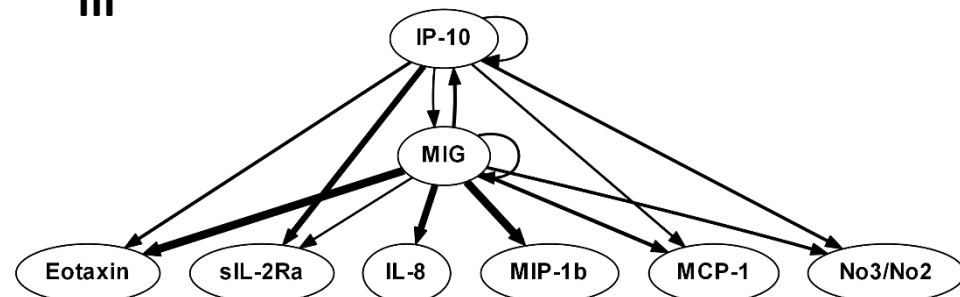

Repeat 2

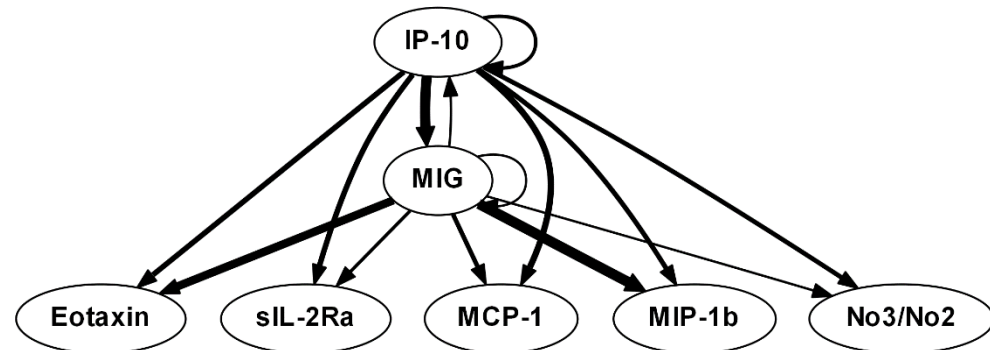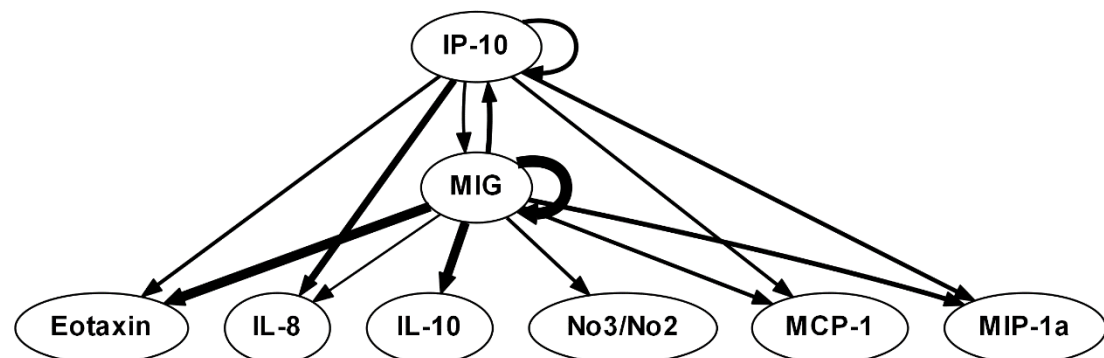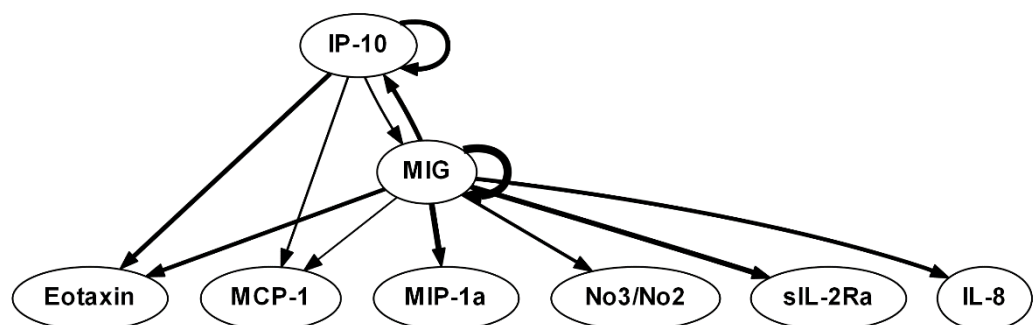

Repeat 3

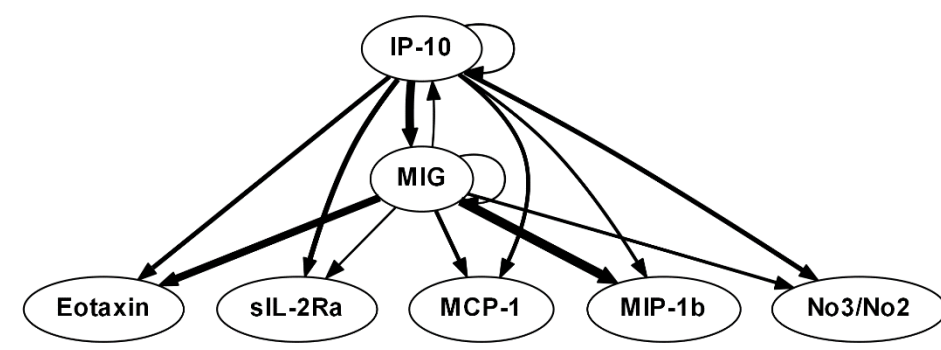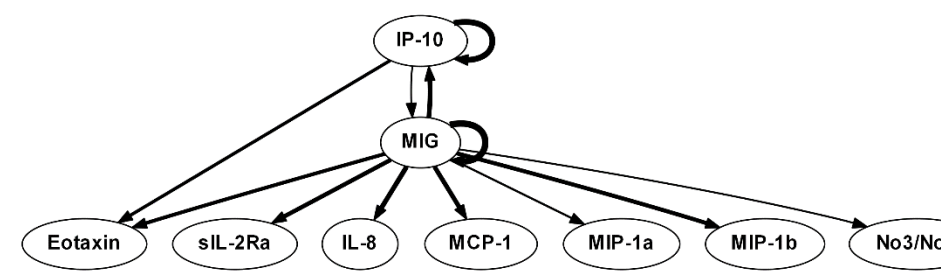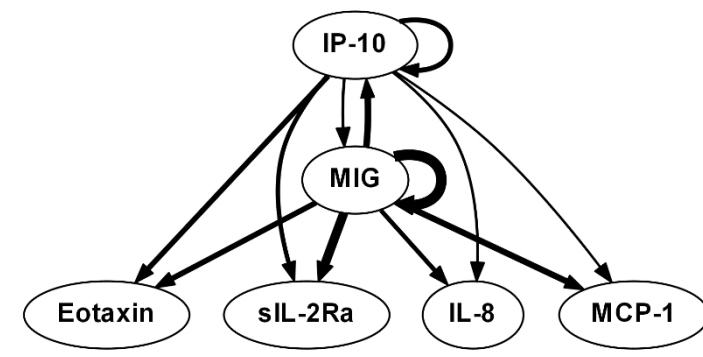

Supplement: Figure S4 — DBN results from randomized outcome groups. Patients were grouped randomly into three groups of sizes of 27, 15, and 7 while maintaining approximately the same percentage of SS (55%):NS (14%):LTx (31%) in each group (panel A) or allowing the percentages to vary (panel B). DBNs were inferred on each group and showed no major differences, with the core module of IP-10 and MIG self-feedback and cross-regulation being observed in all networks. Groups I, II, and III have 15, 27, and 7 patients respectively. (PDF) [file pone.0078202.s004.pdf]

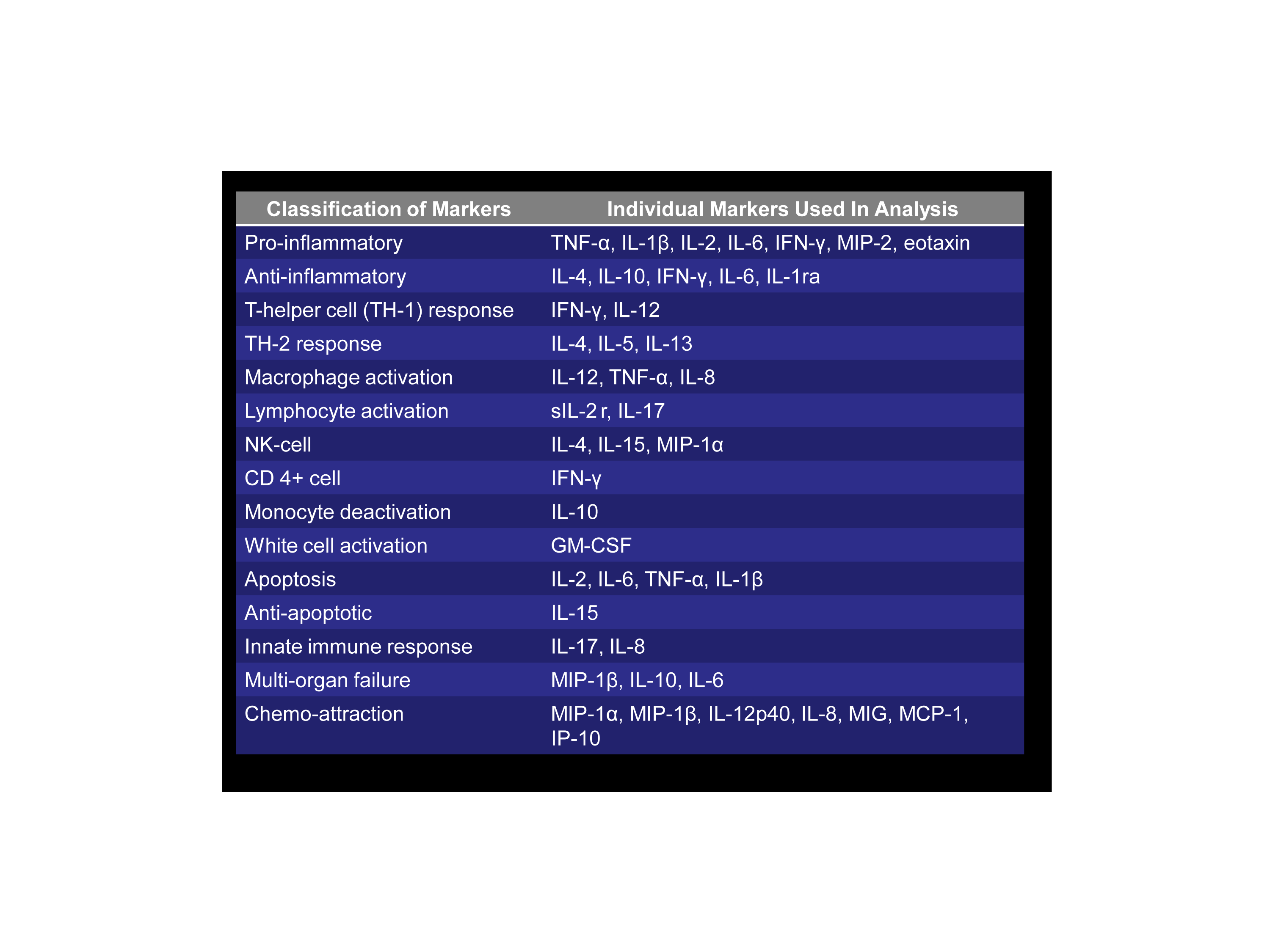

Supplement: Table S1 — List of inflammatory mediators assayed in patient serum. (TIF) [file pone.0078202.s005.tif]
