# Supplementary material for: Analysis of Serum Inflammatory Mediators Identifies Unique Dynamic Networks Associated with Death and Spontaneous Survival in Pediatric Acute Liver Failure
Source: PLoS One. 2013 Nov 11;8(11):e78202. doi: 10.1371/journal.pone.0078202 (PMC3823926; doi:10.1371/journal.pone.0078202)
Supplement: Materials S1 — List of the Institutional Review Boards from all of the participating institutions. (DOCX) [file pone.0078202.s006.docx]

During the period of this study, the PALF study group consisted of 22 pediatric sites: 19 centers : 19 centers in the United States, one in Canada, and two in the United Kingdom. Patient enrollment began in December 1999. The study was approved by the Institutional Review Boards from all of the participating institutions:

1. Institutional Review Board for Baylor College of Medicine and Affiliated Hospitals

2. King’s College Hospital Research Ethics Committee

3. Children’s Memorial Hospital Institutional Review Board

4. Children’s Hospital of Philadelphia Institutional Review Board

5. University of Pittsburgh Institutional Review Board

6. Cincinnati Children’s Hospital Medical Center Institutional Review Board

7. Colorado Multiple Institutional Review Board

8. Columbia University Medical Center Institutional Review Board

9. Emory University Institutional Review Board

10. Committee on Clinical Investigation, Children’s Hospital, Boston

11. Johns Hopkins Medicine Institutional Review Board

12. Indiana University Institutional Review Board

13. University of Michigan Medical School Institutional Review Board

14. Institutional Review Board of the Mount Sinai School of Medicine

15. Sick Kids Research Ethics Board

16. UCSF Human Research Protection Program Committee on Human Research

17. UT Southwestern Institutional Review Board

18. Seattle Children’s Institutional Review Board Subcommittee

19. Washington University in St. Louis Institutional Review Board

In addition, the NIH provided a Certificate of Confidentiality to the study. Written informed consent was obtained from the parents or guardians of the children in the study.
