# Supplementary material for: Analysis of Serum Inflammatory Mediators Identifies Unique Dynamic Networks Associated with Death and Spontaneous Survival in Pediatric Acute Liver Failure
Source: PLoS One. 2013 Nov 11;8(11):e78202. doi: 10.1371/journal.pone.0078202 (PMC3823926; doi:10.1371/journal.pone.0078202)
Supplement: Materials S2 — Detailed description of PCA, hierarchical clustering, and DBN methods. (DOCX) [file pone.0078202.s007.docx]

**SUPPLEMENTARY METHODS**

*Details of patient-specific Principal Component Analysis [PCA].* The goal of this analysis was to identify subsets of mediators that were most strongly correlated with the inflammatory response trajectory of a given PALF patient. To accomplish this, we utilized PCA to determine the dominance of each mediator relative to all other measured mediators that accounted for a given patient’s inflammatory response during the seven days from which the serum samples were taken. A minimum of 3 samples were available for cytokine analysis for each patient during the seven days following enrollment into the PALFSG. First, serum sample underwent cytokine measurements that were then normalized so that all cytokine levels were converted into the same range (from 0 to 1). Next, PCA was computed utilizing the normalized cytokine results derived from all samples that were collected for each patient. A PCA score was calculated for each cytokine, summarizing the relative degree to which that cytokine contributed to the inflammatory response for that patient over time. This was calculated by scaling each principal component’s eigenvector by its respective eigenvalue and summing together the coefficients (loadings) that correspond to a given cytokine over all eigenvectors (sufficient to capture at least 95% of the variance in the data). These PCA scores taken together made up a patient’s “inflammation barcode.” This barcode was used to group patients using hierarchical clustering as described below. Resultant patient sub-groups were then cross-correlated with clinical outcomes: spontaneous survivor, non-survivor with native liver, or received liver transplant.

*Details of hierarchical clustering analysis*. The goal of this analysis was to highlight the natural variability, as well as any overlap, in inflammatory mediators from PALF patients that survived spontaneously without LTx, that died without receiving LTx, or that received LTx. Hierarchical clustering is a simple and unbiased method for segregating series of numerical values by similarity to each other. The limitation of this analysis is that the cluster must be built pairwise; since it is purely based on the similarity between the data, and the cluster may lack biological relevance(1). This analysis was performed for all the inflammatory analytes – both unprocessed and following the patient-specific PCA described above – as published previously(2). Each row of the data matrix corresponds to either a single sample from patient (in raw data clustering) or a single patient’s “inflammation barcode” derived from PCA clustering (see above), and each column corresponds to anti-inflammatory analyte (26 total: 25 cytokines/chemokines along with NO_2_^-^/NO_3_^-^). The magnitudes of these values were log-transformed and indicated by colors. The dendrogram (a branching diagram used to show relationships between members of a group) on the y-axis shows the similarities among samples according to their correlation measures (the correlation between the inflammatory mediators profiles) across all analyte values. The calculation is performed by using the Bioinformatics Toolbox in Matlab® 7.6.0, and the code for this algorithm has been made available publicly (2).

Luminex technology affords us the advantage of making many cytokine measurements from the same biological sample. Our dataset consists of 215 serum samples, each yielding 26 mediator measurements. We consider each set of mediator measurements belonging to the same serum sample to be a single point in 26-dimensional cytokine space. We used the Euclidean distance between these points in 26-dimensional space as the distance metric in our hierarchical clustering scheme. When clustering these raw data, we took each of the 215 points to be independent, even though several of them came from the same patient (median = 4 samples/ patient). Because each serum sample was independent, there was no guarantee that all samples from a given patient would cluster together. This led to the possibility that two measurements from the same patient could be assigned to different clusters. We examined the two clusters resulting from the first separation in the dendrogram (see Fig. 1), and found that samples from the same patient were rarely segregated into opposite clusters, For 32 out of 49 total patients, all serum samples clustered together. Of the 17 remaining, there were only 4 patients whose data points were split equally between the two clusters. Of the 13 patients whose data were segregated unequally, only two patients had more than one data point in their minority cluster. Relabeling those points as each patient’s majority cluster did not lead to a significantly different clustering (adjusted rand index was 0.59, where perfect agreement yields rand index = 1, no agreement = 0(3)). The resultant clusters also showed no strong correlation with clinical outcomes (adjusted rand index was 0.023). From this analysis, we concluded that unsupervised clustering of raw cytokine measurements was incapable of predicting clinical outcomes for these patients.

*Details of Dynamic Bayesian Network analysis.* Time courses of cytokine measurements from each experiment were used as input for a Dynamic Bayesian Network (DBN) inference algorithm. The program was implemented in Matlab® and has been described previously by others (4). A number of leave-one-out inference procedures were performed on the merged data to obtain a measure of the robustness of inferred interactions. Briefly, the algorithm uses an inhomogeneous dynamic changepoint Bayesian Gaussian with score equivalence (BGe) model that allows the reconstruction of time-varying DBNs. In this study, we chose to focus on a static network that describes the entire time-course. For each node, a new set of parent nodes was sampled directly from the posterior distribution and the local scores computed using the BGe model. Each node was subject to a fan-in restriction of three parent nodes. The Gibbs sampling procedure was run for 100 steps to yield a final network structure. Individual networks were then averaged to obtain a consensus network for each condition according to the following rule: if a particular edge was present in more than 50% of the individual networks in a particular condition, it was included in the consensus network, otherwise it was excluded. We note that the program is probabilistic and thus applying this hard cutoff can lead to minor differences in results from different runs. However, upon multiple runs on different machines, we observed the same core network structure using this method with at most two peripheral nodes being different.

Individual patient data were time-shifted and merged in a mediator (node)-specific manner, yielding a matrix with *n* time courses for each mediator, where *n* is the number of patients in a particular group (survivors, non-survivors, or LTx). The node-specific mixtures of latent variables were chosen from the entire set of time series for that node. Thus, the BGe score, and consequently, the marginal posterior probabilities of the edges in the final network structure, were reflective of the distribution of the data across time as well as across patients. This analysis was then repeated *n* times, excluding one patient at a time from the merged dataset, and thus obtaining *n* different networks. The marginal posterior probabilities were then averaged over these networks, and reported as an indicator of the confidence in the inferred interactions. Only edges with marginal probabilities greater than 0.5 were included in the reported network structure.

References

1. Janes KA, Yaffe MB. Data-driven modelling of signal-transduction networks. Nat Rev Mol Cell Biol 2006 Nov;7(11):820-828.

2. Mi Q, Constantine G, Ziraldo C, Solovyev A, Torres A, Namas R, et al. A dynamic view of trauma/hemorrhage-induced inflammation in mice: Principal drivers and networks. PLoS ONE 2011;6:e19424.

3. Hubert L, Arabie P. Comparing partitions. Journal of Classification 1985 Dec 1;2(1):193-218.

4. Grzegorczyk M, Husmeier D. Improvements in the reconstruction of time-varying gene regulatory networks: dynamic programming and regularization by information sharing among genes. Bioinformatics 2010 Dec 21.
